# A Gustatory Receptor GR8 Tunes Specifically to D-Fructose in the Common Cutworm *Spodoptera litura*

**DOI:** 10.3390/insects10090272

**Published:** 2019-08-26

**Authors:** Xiao-Long Liu, Qi Yan, Yi-Lin Yang, Wen Hou, Chun-Li Miao, Ying-Chuan Peng, Shuang-Lin Dong

**Affiliations:** 1 Key Laboratory of Integrated Management of Crop Disease and Pests, Ministry of Education/Department of Entomology, Nanjing Agricultural University, Nanjing 210095, China; 2 Institute of Entomology, Jiangxi Agricultural University, Nanchang 330045, China

**Keywords:** gustatory receptor, *Spodoptera litura*, D-fructose, *Xenopus* oocyte, proboscis extension reflex

## Abstract

Gustatory receptors (GRs) are crucial in the peripheral coding of the non-volatile compounds in insects, and thus play important roles in multiple behaviors including feeding, mating, and oviposition. However, little research has been done on GRs in lepidopteran pests. In the current work with *Spodoptera litura*, an important worldwide crop’s pest, a candidate fructose GR gene (*SlitGR8*) was cloned in full length, and its spatial and temporal expression profiles were determined by quantitative real-time PCR (qPCR). It revealed that *SlitGR8* was highly expressed in antennae of both male and female adults, as well as in larva of first, fifth and sixth instar. Functional analyses were further conducted using the *Xenopus* oocyte system. *SlitGR8* responded specifically to D-fructose among 12 tested sugar compounds. In addition, the behavioral assay demonstrated that both female and male moths could respond with proboscis extension behavior to D-fructose applied onto the antenna, but females showed higher sensitivity than males. The results provide an important base for further elucidation of molecular mechanisms of gustation, and a potential target for development of feeding interfering technique in *S. litura*.

## 1. Introduction

Insect acclimatization to their chemical environments and survival are mainly dependent on their complex and specialized chemosensory system, particularly in the moth species. Male moths use their olfaction to perceive female sex pheromones and to locate mates, while female moths use both the olfaction and gustation in searching for egg-laying sites. Caterpillars use the system for locating, identifying, and evaluating potential foods by discriminating nutritious chemicals from harmful or toxic compounds [1]. Gustatory sensilla in insects are broadly distributed on the mouthparts, antennae, wings, legs, and ovipositor [2,3,4].

The origin of the gustatory receptor (GR) genes potentially dates back to the non-insect arthropods [5,6]. GR proteins have the signature seven-transmembrane domain topology [7,8]. The general process of insect gustation at the peripheral level has been suggested to include several major steps [9]. First, soluble chemicals enter into the sensilla cavity through the pore and begin contacting the dendrite of the gustatory receptor neurons (GRNs). Then, GRs on the dendrite are activated by the chemical molecules, leading to generation of electrical signals. After that, electrical signals are transmitted to the central nervous system through nerve axons in the form of pulses.

GRs can function independently as monomers, such as the sugar receptor [10,11], or form obligatory heteromers of two receptors as the CO_2_ receptor [12,13] or sugar receptor [14], unlike odorant receptors (ORs) that are functional only in the form of heteromers with the odorant receptor coreceptor (Orco) [7,15]. In contrast to the large number of ORs that are functionally characterized, only few GRs have been functionally documented. In *Drosophila melanogaster*, GR43a [11] and GR64f combination with GR5a [14] responded specifically to D-fructose and trehalose, respectively, while GR64f concert with GR64a responded to multiple sugar components [16,17]. In *Bombyx mori*, GR8 responded to *myo*-inositol and *epi*-inositol [8] and GR10 had same response [18]. GR9 in *B. mori* [10] and GR4 in *Helicoverpa armigera* [19] are orthologs and were characterized as specific receptors of D-fructose, while GR9 in *H. armigera* responded to D-galactose and D-maltose, in addition to D-fructose [20], indicating a functional divergence with its paralog of *H. armigera* GR4. To clarify the evolution and functional divergence, characterizations of D-fructose receptors in more moths are needed.

The tobacco cutworm *Spodoptera litura* (Lepidoptera, Noctuidae), a polyphagous and serious agricultural pest worldwide, causes huge crop losses each year [21]. D-fructose receptors play important roles in food evaluation and feeding for both larval and adult *S. litura*. However, no study has addressed this topic to date. Here, we reported the characterization of a D-fructose specific sugar receptor, *SlitGR8*, in *S. litura.* The cDNA sequence was cloned and analyzed in relation to GR genes from other representative insect species, the spatial and temporal expression profiles were determined by the quantitative real-time PCR (qPCR), and finally, the function was elucidated by the heterologous expression system of *Xenopus* oocyte with two-electrode voltage-clamp physiological recordings. The results are helpful for understanding the gustatory mechanisms and for developing GR-based feeding interfering techniques in adult and larval *S. litura*.

## 2. Materials and Methods

### 2.1. Insect Rearing and Tissue Collection

The *S. litura* larvae were fed an artificial diet in the laboratory in 14 L:10 D at 26 ± 1 °C and 65 ± 5% relative humidity [22]. Pupae were separated by sex and placed in separate cages (20 cm × 20 cm × 30 cm). After the pupae emerged, the moths were fed with 10% honey solution.

To determine the gene expressions at different developmental stages, 300 eggs, 300 fist, 300 second, 150 third, 90 fourth, 60 fifth, and 60 sixth instar larvae, 60 male and 60 female pupae, 60 male and 60 female adults were collected, respectively. To determine the gene expressions at different adult tissues, the tissues (90 pairs of antennae, 90 probosces, 90 pairs of labial palpi, 60 heads, 30 thoraxes, 30 abdomens, 240 legs, and 60 pairs of wings) were collected. The numbers represent three replicates of each developmental stage or tissue were collected. The collected tissues were immediately frozen in liquid nitrogen and stored at −80 °C until use for RNA extraction.

### 2.2. Sugar Compounds

The 12 tested sugars (*epi*-inositol, D-arabinose, D-raffinose, D-galactose, *myo*-inositol, D-maltose, D-glucose, D-sucrose, D-fructose, D-mannose, D-trehalose, and D-cellobiose) were all purchased from J&K scientific (purity ≥ 96%), Beijing, China. Stock solutions (1 M) were prepared using ND96 buffer (100 mM NaCl, 2 mM KCl, 1.8 mM CaCl_2_, 1 mM MgCl_2_, 5 mM HEPES, pH 7.6). Prior to experiments, the stock solutions were diluted into ND96 buffer to obtain the testing concentrations. All compounds were freshly prepared for the experiments.

### 2.3. RNA Isolation and cDNA Synthesis

Total RNA was extracted using Trizol Reagent (Invitrogen, Carlsbad, CA, USA) following the manufacturer’s instructions. The quality of RNAs was checked with NanoDrop-2000 (Thermo Scientific, Waltham, MA, USA) following the standards of 260/280 = 1.8–2.0, and 260/230 = 2.0–2.5. The first single strand cDNAs were synthesized from 1μg total RNA using HiScript^®^ III RT SuperMix for qPCR (+gDNA wiper) (Vazyme, Nanjing, China) according to the provided protocol.

### 2.4. Gene Cloning and RACE Amplification

There were two reported full length *SlitGR8s*, with one in NCBI (XM_022976219.1) and another by the paper [23]. The two *SlitGR8s* were 1425 bp and 1413 bp, encoding 474 and 470 amino acid residues, respectively. These two *SlitGR8s*, which share high identity with D-fructose receptors in other moth species (Table 1), were regarded as the potential D-fructose receptors in *S. litura.* End-to-end PCRs were firstly conducted to verify these two *SlitGR8s,* but failed to yield any of the two *SlitGR8s*. Then, PCRs were conducted to clone the partial sequence using primers based on the consensus part of the two *SlitGR8s*, and 5′RACE was further conducted to obtain the full-length *SlitGR8* sequence. All primers were designed by Primer Premier 5.0 (PREMIER Biosoft International, CA, USA) (Table S1). The PCR reaction was performed in 25 μL containing 12.5 μL of 2× Phanta Max Master Mix, 9.5 μL of ddH_2_O, 1 μL of cDNA template, and 1 μL of each primer (10 μM). The PCR conditions were 95 °C for 3 min; 34 cycles of 95 °C for 15 s, 55 °C for 15 s, 72 °C for 90 s; 72 °C for 5 min. PCR products were run on a 1.2% agarose gel, and the band was recovered and purified by AxyPrep™ DNA Gel Extraction Kit (Axygen, Suzhou, Jiangsu, China). Purified PCR produces were cloned into the vector pEASY-Blunt T3 (TransGen Biotech, Beijing, China), and positive clones were sequenced by the company (Tongyong, Anhui, China).

The 5′RACEs were conducted to obtain the full-length sequence, using the SMART™ RACE cDNA Amplification Kit (Clontech, Mountain View, CA, USA) according to the manufacturer’s instructions. All parameters were same as those in the manufacturer’s instructions. The full-length *SlitGR8* sequence was determined by assembling the cDNA fragments from the 5′ RACEs and confirmed by the end-to-end PCR.

### 2.5. Sequence Analysis and Phylogenetic Tree Construction

Transmembrane domains of the GR’s amino acid sequences were predicted by TMHMM Server Version 2.0 (http://www.cbs.dtu.dk/services/TMHMM). The protein sequences data were aligned and compared with DNAMAN 5.2.2 (Lynnon BioSoft., San Ramon, CA, USA). The secondary structure predicted by TOPCONS (topcons.net) models [24]. For the phylogeny, SlitGRs were clustered with GRs from 3 representative species were all reported to be functionally characterized of *D. melanogaster* [5,6,11,14,17], *B. mori* [8,10,20,25], and *H. armigera* [18,26]. The protein sequences were aligned with the MAFFT (v. 7.313, E-INS-I parameter). Phylogenetic trees were constructed using the neighbor-joining method with 1000 bootstrap resampling by MEGA5.0 [27]. 

### 2.6. Spatial and Temporal Expression Analysis

Total RNA was extracted, and cDNA templates were synthesized as mentioned above. The qPCR specific primers (Table S1) were designed by Beacon Designer 8.0 (PRIMER Biosoft International, CA, USA). The qPCR was performed on QuantStudio™ 6 Flex Real-Time PCR System (Applied Biosystems, Foster City, CA, USA) with ChamQ^TM^ Universal SYBR^®^ qPCR Master Mix (Vazyme, Nanjing, China). Each 20 μL reaction containing 10 μL of 2× ChamQ Universal SYBR qPCR Master Mix, 0.4 μL of each primer (10 μM), 2 μL of cDNA template and 7.2 μL of nuclease-free water. The cycling parameters were: 95 °C for 30 s, 40 cycles of 95 °C for 5 s, and 60 °C for 34 s. The housekeeping genes glyceraldehyde-3-phosphate dehydrogenase (*GAPDH*) and elongation factor 1α (*EF1α*) were used as references to normalize the target gene expression. Each reaction was run in three technical replicates and the means and standard errors were obtained from three independent biological replicates. Gene expression levels were analyzed using the 2^−ΔΔCT^ method [28]. 

### 2.7. Vector Construction and cRNA Synthesis

Primers that containing homologous recombination sequence and restriction enzymes (*EcoRI* and *XbaI*) were designed to amplify the ORFs of *SlitGR8* (Table S1). The confirmed plasmid *SlitGR8* and PGH19 expression vector plasmid were homologously recombined with ClonExpress One Step Cloning Kit (Vazyme Biotech Co., Ltd.). The plasmids were extracted by miniprep method with Plasmid Mini Kit Ι (Omega, Bio-Tek) and were purified with hydroxybenzene-chloroform-Isoamyl alcohol. The purified plasmid was linearized by restriction enzyme (*NdeI*) at 37 °C overnight and used as templates to synthesize cRNA using T7 polymerase of mMESSAGE mMACHINE T7 Kit (Invitrogen). The reaction containing 2 μL 10× Reaction Buffer, 10 μL 2× NTP/CAP, 1 μg linearized plasmid, 2 μL Enzyme Mix, and Nuclease-free water up to 20 μL was conducted at 37 °C for 2 h. The cRNA was diluted with nuclease-free water at a concentration of 2000 ng/µL and stored at −80 °C until use. 

### 2.8. Receptor Expression in Xenopus Oocytes and Electrophysiological Recordings

*SlitGR8* was expressed in *Xenopus* oocytes, and detected for the ligand sensitivity using two electrode voltage-clamp recordings, following reported protocols [29,30]. *Xenopus laevis* was anesthetized by immersing it in ice for 20–40 min. The oocytes were dissected freshly from *Xenopus.* Mature healthy oocytes (stages V–VII) were treated with collagenase type 1A (2 mg mL^−1^) in Ca^2+^-free buffer (100 mM NaCl, 2 mM KCl, 1 mM MgCl_2_, and 5 mM HEPES, pH 7.6) for 1–2 h at 18 °C. Each oocyte was microinjected with 27.6 ng of GR8 cRNA. Then, the oocytes were incubated at 18 °C for 3–5 days in ND96 solution, supplemented with dialyzed horse serum (5%) and 2.5 mM sodium pyruvate for electrophysiological recording. 

Electrophysiological responses were recorded from the injected *Xenopus* oocytes using a two-electrode voltage-clamp setup on the Axoclamp 900 A Microelectrode Amplifier plate (AutoMate Scientific Inc., San Jose, CA, USA) at a holding potential of −80 mV. Two micropipettes were prepared from glass capillaries using the Flaming/Brown Micropipette Puller (P-97, Sutter Instrument, Novato, CA, USA). Oocytes were exposed to sugar compounds, with an interval between exposures that allowed the current to return to baseline. The current signals were digitized by the Axon Digidata 1440 A Data Acquisition System (AutoMate Scientific, Inc., Union City, CA, USA). CLAMP 10.4 software (Axon Instruments Inc., Union City, CA, USA) was used to analyze the data. Dose–response data were analyzed using GraphPad Prism 7 (GraphPad Software, Inc., San Diego, CA, USA).

### 2.9. Proboscis Extension Reflex (PER) Assay

The two-day-old female and male adults were fed with double distilled water until they stopped feeding (as moths no longer extend proboscis to water provided), followed by starvation for 24 h. To do the assay, a moth was restrained in a 1.5 mL EP tube, allowing the head exposed by cutting the edge of the top. The swab tip with fructose or control solution was carefully touched onto the distal part of moth antennae, and proboscis extension reflex (PER) was recorded within 30 s of the stimulation. Double distilled water was used as the control and solvent. Four concentrations (0.001 M, 0.01 M, 0.1 M, and 1 M) of fructose solution were used for the test. Twenty individual moths were tested for each concentration. The assays were conducted in three replicates. 

## 3. Results

### 3.1. Gene Cloning and Phylogenetic Analysis

Sequence analysis showed that among all SlitGRs, the two *SlitGR8s* shared highest identity with reported fructose GRs from *H. armigera* and *B. mori* (Table 1), suggesting the two *SlitGR8s* being potential fructose GRs in *S. litura.* These two *SlitGR8s* were different at 5′ terminus (Figure S1). To verify the two *SlitGR8* sequences, PCRs were conducted using specific primers, but failed to obtain any PCR product, indicating mistakes in the two sequences. Then, 5′ primer for the consensus part of the two *SlitGR8* was used, and a partial sequence was successfully obtained. With this partial sequence, a full-length sequence was further obtained by 5′RACE and end-to-end confirmation, which consisted of 1497 bp, encoding 498 amino acid residues. This sequence was hereafter named *SlitGR8* and used for further study.

**Figure 1:**
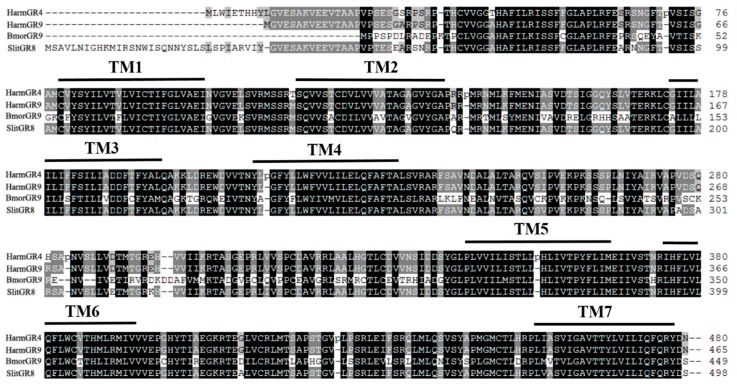
Alignment of the amino acid sequences of four lepidopteran fructose GR genes, showing positions of seven predicted transmembrane domains (**TM1**–**TM7**). Identical amino acids are marked with black shading. Insect species and GenBank IDs of the GR genes are same as in Table 1.

**Figure 2:**
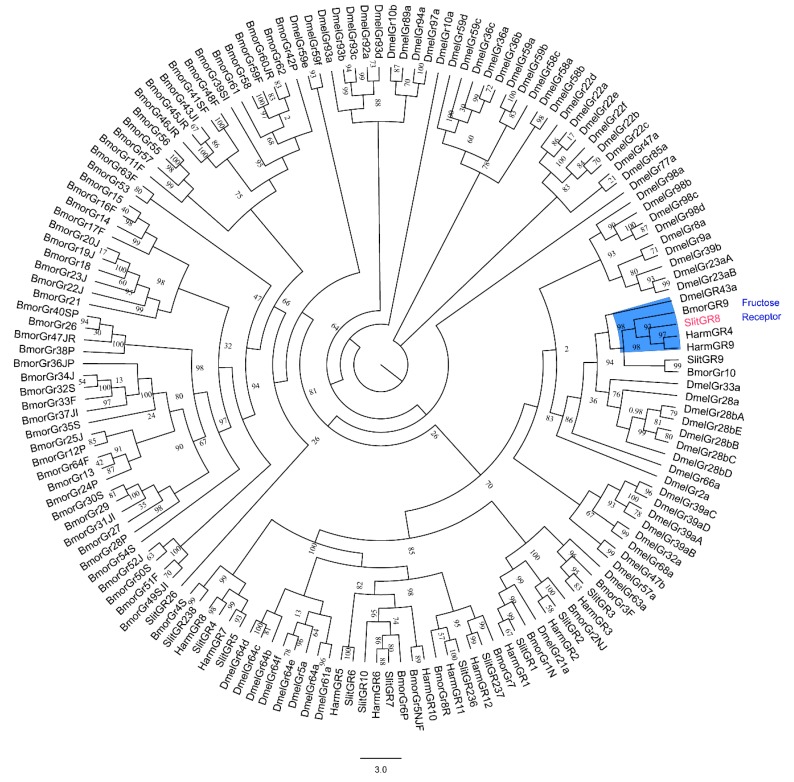
Phylogenetic tree of the amino acid sequences of *SlitGR8* and other GRs, including all functionally characterized fructose GRs from other species of *D. melanogaster*, *B. mori*, and *H. armigera*. The tree was constructed by neighbor joining method with a bootstrap of 1000. Fructose receptor clad is marked in blue color. *SlitGR8* is in red color.

Like other insect GRs, *SlitGR8* contains seven putative transmembrane domains (Figure 1). *SlitGR8* shared high identity with fructose GRs from lepidopteran species *H. armigera* (HarmGR9, 89.8%) and (HarmGR4, 87.3%), while low identity was shared with fructose GRs from *D. melanogaster* (DmelGR43a, 23.3%) in Diptera (Table 1). In agreement, phylogenetic tree showed that *SlitGR8* clustered together with fructose GRs of other lepidopteran insects and DmelGR43a of *D. melanogaster* (Figure 2). 

### 3.2. Spatial and Temporal Expression of SlitGR8 

**Figure 3:**
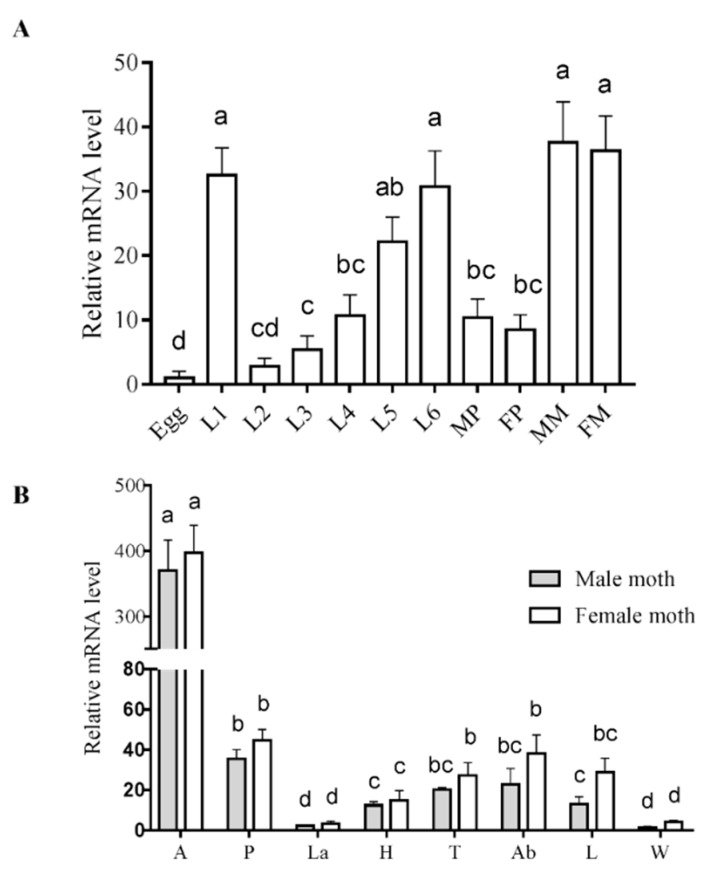
Relative expression levels of *SlitGR8* mRNA at different development stages (**A**) and in different tissues (**B**). (**A**) L1–L6: 1–6 instar larva; MP: Male pupae; FP: Female pupae; MM: Male moths; FM: Female moths. (**B**) A: Antennae; P: Proboscis; La: Labial palpi; H: Heads; T: Thoraxes; Ab: Abdomens; L: Legs; W: Wings. Different lowercase letters indicate significant differences (*p* < 0.05) based on one-way ANOVA, followed by Tukey’s HSD test for multiple comparisons. Means± standard error from three replicates are shown.

Analysis of expression at different developmental stages revealed that *SlitGR8* was expressed at all life stages and was higher in the first, fifth, sixth instar larvae and moths, but lower in eggs (Figure 3A). Tissue expression profile showed that *SlitGR8* were highly expressed in antennae without obvious sex bias, while it was also expressed considerable levels in proboscises, abdomens, and legs (Figure 3B).

### 3.3. Functional Characterization of SlitGR8

**Figure 4:**
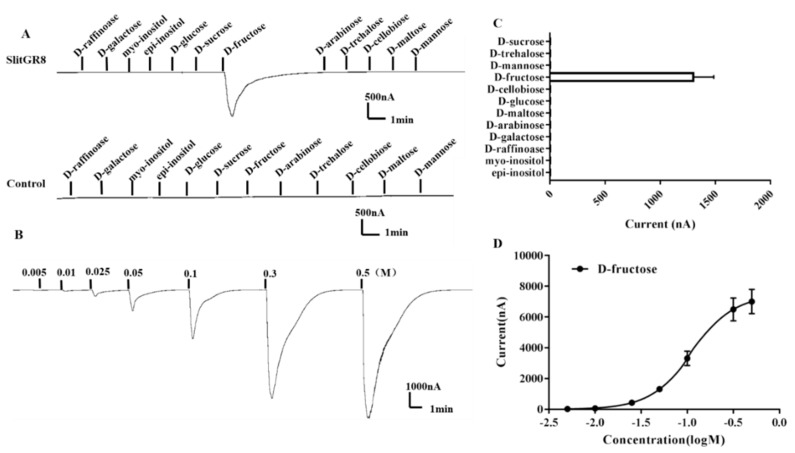
Responses of *Xenopus* oocytes expressed with *SlitGR8* to stimulation of sugar components. (**A**) Inward currents of *Xenopus* oocytes injected with *SlitGR8* cRNA (upper) and buffer (lower) to sugar components (0.1 M). (**B**) Response profile of *Xenopus* oocytes expressed with *SlitGR8*. Error bars indicate SEM (n = 5). (**C**) Response of *SlitGR8* expressing *Xenopus* oocytes to D-fructose of different concentrations. (**D**) Dose–response curve of *SlitGR8* expressing *Xenopus* oocytes to D-fructose, with EC_50_ = 0.217 M. Error bars indicate SEM (n = 5).

*Xenopus* oocyte expression and the voltage clamp recording system was used to characterize the function of *SlitGR8*. Twelve sugars at a concentration of 0.1 M was tested to determine the ability in triggering the responses of *SlitGR8*. The results showed that *SlitGR8* exclusively responded to D-fructose (Figure 4A,B). Further dose-response study was conducted using a range of fructose concentrations (0.005 M, 0.01 M, 0.025 M, 0.05 M, 0.1 M, 0.3 M, and 0.5 M). Results showed that the oocytes displayed detectable responses to D-fructose at the initial concentration of 0.005 M (24.25 ± 4.78 nA), and the response reached plateau around 0.3 to 0.5 M (Figure 4C,D). The EC_50_ value of D-fructose was calculated to be 0.217 M. Oocytes injected with buffer alone (control) showed no responses to any of the sugars.

### 3.4. Proboscis Extension Reflex (PER) Induced by D-Fructose

**Figure 5:**
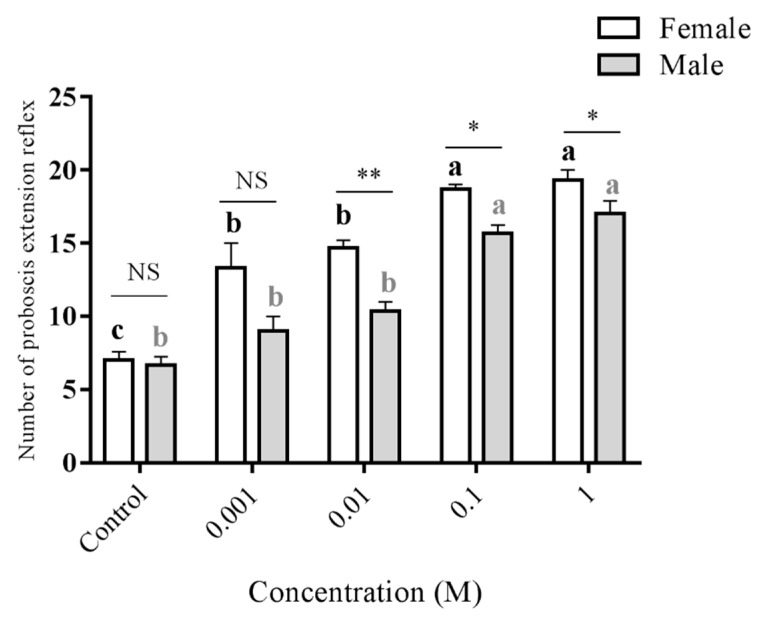
Proboscis extension reflex (PER) resulted from adult male and female antennae stimulation by 0.001 M, 0.01 M, 0.1 M and 1 M fructose. Double distilled water (the solvent) was used as the control. Bars represent standard error form three replications. Different letters (a–c) indicate significant differences (*p* < 0.05) of male or female responses among different concentrations based on one-way ANOVA followed by Tukey’s HSD test for multiple comparisons. * and ** mean significant differences (*p* < 0.05 and <0.01, respectively), while NS indicates no significant difference between male and female responses by T-test.

To confirm the ability of antennae in detecting D-fructose, and further, in stimulating moth feeding, PER test was conducted. By touching the distal part of antennae with D-fructose of range of concentration (0.001 M, 0.01 M, 0.1 M, and 1 M), significant PER was observed in females for all tested concentrations, while significant PER in males was only observed at the highest two concentrations (Figure 5). The results indicated that both female and male adults can detect D-fructose by antennae to elicit feeding behavior, but females are more sensitive than males.

## 4. Discussion

Gustatory systems play a critical role in insect multiple behaviors, including feeding, mating, and oviposition. In the present study, we functionally identified a D-fructose specific GR in the polyphagous pest *S. litura*, providing an important base for understanding molecular mechanisms of the gustation in moth species. 

Two *SlitGR8* cDNA sequences with complete ORF have been reported, with one in NCBI and another in a paper [23]. Both *SlitGR8s* have a seven-transmembrane domain topology, which is in accordance with the other insect GRs [7,25], and share high identity with the reported D-fructose specific GRs in *B. mori* and *H. armigera*. Noticing the difference between the two sequences at the 5′ end, we tried to confirm it by PCR amplification, but unfortunately, we failed to obtain each of the two sequences. This suggests that the two reported sequences might be incorrect, possibly due to mistakes in the sequence annotation. The *SlitGR8* cloned by the present study clarifies the *SlitGR8* sequence, allowing us to conduct functional study. 

D-fructose is a main sugar component in floral nectars [31,32,33]. Our *Xenopus* oocyte expression-based functional study showed that *SlitGR8* specifically responded to D-fructose but did not respond to 11 other sugar compounds. It is reasonable that major compounds are detected by ligand specific GRs, although it may also be detected by GRs with a wide ligand range [19]. In addition, our results revealed that *SlitGR8* functions alone, which is same as other insect GRs in the fructose clade, but is unlike mammalian sugar receptors that function in heterodimers [34] and insect ORs that function always in combination with the odorant receptor coreceptor (Orco). Nevertheless, some studies suggested that the co-expression of GRs is necessary for detection of sugars in insects. For instance, Gr64f was required to combine with Gr5a for the behavioral response to trehalose, and Gr64f was in concert with Gr64a to rescue the defects in the sensitivities to sucrose, maltose and glucose in *Drosophila* [14]. This disagreement indicates insect GRs is more complex than ORs regarding the functional combination of different receptors.

The moths swing their antennae actively not only to smell odorants, but also to detect sugars and other compounds [35,36]. Our behavioral bioassay demonstrated that D-fructose can induce the proboscis extension in both females and males when antennae are in contact with the sugar solution. Similar results were also reported in female *H. armigera* [18]. The proboscis extension assay and the high expression levels of *SlitGR8* in antennae (ten-fold and more than in other tissues) clearly suggest that antennae are a major tissue for detection of D-fructose in *S. litura*. Furthermore, female moths showed higher sensitivity than males for D-fructose, which is consistent with the fact that females feed more than males for ovary maturation and egg production and development. However, the *SlitGR8* expression levels are similar between female and male moths, suggesting that the difference in D-fructose sensitivity may result from events in central nervous system. Besides of adult moths, caterpillars also use sugars as major nutrients and often as phagostimulants [37]. Our qPCR analysis using the whole larvae showed that *SlitGR8* was also expressed in the larvae, particularly in first, fifth, and sixth instar larvae. The neonates are generally more sensitive to environmental stimulation including food signals, and thus have a high expression of *SlitGR8*. For the honeybee (*Apis mellifera*), the high-instar larvae prefer the food containing more fructose than low-instar larvae [38], and similarly, in the cotton bollworm *H*. *armigera*, high-instar larvae prefer to feeding on plant tissues containing more sugar nutrients [39]. The high *SlitGR8* expression in the fifth and sixth instar larvae suggests that D-fructose plays important role in the feeding and food selection in the high instar larvae for *S. litura*, which needs to be verified by further functional analysis. The overall high expression in the larvae provides a preferable opportunity for development of the feeding interfering technique targeting at *SlitGR8*. 

In conclusion, we cloned a full-length *SlitGR8* gene by checking and correcting the two reported *SlitGR8* sequences. Further phylogenetic analysis and gene expression profile analysis revealed that *SlitGR8* was enriched in the antennae, and finally, a functional study demonstrated that *SlitGR8* specifically tuned to D-fructose. In addition, proboscis extension reflex test confirmed the ability of adult antennae in detecting D-fructose, and thus eliciting moth’s feeding behavior. These findings will help us better understand the molecular mechanisms of sugar recognition and develop feeding interfering technique in *S. litura*.

## Figures and Tables

**Table 1:** Comparison of the amino acid identity of *SlitGR8* and other functionally characterized fructose gustatory receptor (GR) genes reported from different insects.

	SlitGR8	HarmGR4	HarmGR9	BmorGR9	DmelGR43a
NCBI-SlitGR8	–	93.3%	94.5%	60.1%	23.9%
Paper-SlitGR8	–	91.9%	95.1%	61.1%	24.4%
SlitGR8	–	87.3%	89.8%	57.6%	23.3%
HarmGR4		–	95.0%	59.2%	23.7%
HarmGR9			–	62.2%	24.5%
BmorGR9				–	24.0%
DmelGR43a					–

Insect species and GenBank IDs of the GR genes are: *Spodoptera litura* (SlitGR8), this study; *Bombyx mori* (BmorGR9), XP_012551875.1; *Helicoverpa armigera* (HarmGR4), AGK90011.1; (HarmGR9), AGA04648.1; *Drosophila melanogaster* (DmelGR43a), NP_001036531.1. “NCBI-SlitGR8” and “Paper-SlitGR8” represent the SlitGR8 sequence from NCBI and the paper (Cheng et al., 2017), respectively.

## Supplementary Materials

The following are available online at https://www.mdpi.com/2075-4450/10/9/272/s1, Figure S1: Primers used in the study. Table S1: Alignment of the sequences of two reported SlitGR8s.
