# Supplementary material for: A Gustatory Receptor GR8 Tunes Specifically to D-Fructose in the Common Cutworm Spodoptera litura
Source: Insects. 2019 Aug 26;10(9):272. doi: 10.3390/insects10090272 (PMC6780311; doi:10.3390/insects10090272)
Supplement: Supplementary file 1 [file insects-10-00272-s001.zip › Supplementary data.docx]

**Supplementary data**

**Table S1.** Primers used in the study.

| Primer name | Sequence (5´- 3´) |
| --- | --- |
| Primers for 5′RACE |  |
| SlitGR8-5′GSP | GCATTGCGTGGTCGGTGGAGCGCAT |
| Primers for gene cloning |  |
| SlitGR8-F | ATGAGTGCAGTGCTAAACATTG |
| SlitGR8-R | TCAACTGTCGTATCTTTGGAATT |
| Primers for qPCR |  |
| SlitGR8-qF | GCCATCCTCATCTTCTTCTCTATC |
| SlitGR8-qR | ATTGGTTACTACGTCCCATTCTC |
| GAPDH-qF | CGTGTTCCTGTTGCTAAC |
| GAPDH-qR | CTTGACCTTCTGCTTGATAG |
| EF1α-qF | ACGCTCCCGGACACAGAGAT |
| EF1α-qR | GCTCACGGGTCTGTCCGTTC |
| Primers for cRNA synthesis |  |
| SlitGR8-EcoRI-F | ATTCCCCGGGGATCC***GAATTC***ATGAGTGCAGTGCTAAACATTG |
| SlitGR8-XbaI-R | TCGGCGATCGGGCCC***TCTAGA***TCAACTGTCGTATCTTTGGAATT |

The underlined sequences are homologs to the sequences of PGH19. The bold italic sequences (GAATTC and TCTAGA) are the restriction sites of *EcoRI* and *XbaI*, respectively.

**Fig. S1.** Alignment of the sequences of two reported *SlitGR8s*. “NCBI” and “Paper” represent the sequence from NCBI and the paper (Cheng et al., 2017), respectively. The “GR8” full length sequence was obtained by 5’RACE.
